# SmartWear body sensors for neurological and neurosurgical patients: A review of current and future technologies

**DOI:** 10.1016/j.wnsx.2023.100247

**Published:** 2023-11-04

**Authors:** Nithin Gupta, Varun Kasula, Praveen Sanmugananthan, Nicholas Panico, Aimee H. Dubin, David AW. Sykes, Randy S. D'Amico

**Affiliations:** a Campbell University School of Osteopathic Medicine, Lillington, NC, USA; b Kansas City University College of Osteopathic Medicine, Kansas City, MO, USA; c Lake Erie College of Osteopathic Medicine, Erie, PA, USA; d Department of Neurosurgery, Duke University Medical School, Durham, NC, USA; e Lenox Hill Hospital, Department of Neurosurgery, New York, NY, USA

**Keywords:** Body sensors, Wearable technology, Epilepsy, Stroke, Continuous monitoring, Neurosurgical outcomes, Neurodegenerative disease

## Abstract

**Background/objective:**

Recent technological advances have allowed for the development of smart wearable devices (SmartWear) which can be used to monitor various aspects of patient healthcare. These devices provide clinicians with continuous biometric data collection for patients in both inpatient and outpatient settings. Although these devices have been widely used in fields such as cardiology and orthopedics, their use in the field of neurosurgery and neurology remains in its infancy.

**Methods:**

A comprehensive literature search for the current and future applications of SmartWear devices in the above conditions was conducted, focusing on outpatient monitoring.

**Findings:**

Through the integration of sensors which measure parameters such as physical activity, hemodynamic variables, and electrical conductivity - these devices have been applied to patient populations such as those at risk for stroke, suffering from epilepsy, with neurodegenerative disease, with spinal cord injury and/or recovering from neurosurgical procedures. Further, these devices are being tested in various clinical trials and there is a demonstrated interest in the development of new technologies.

**Conclusion:**

This review provides an in-depth evaluation of the use of SmartWear in selected neurological diseases and neurosurgical applications. It is clear that these devices have demonstrated efficacy in a variety of neurological and neurosurgical applications, however challenges such as data privacy and management must be addressed.

## Funding

The authors declare that no funds, grants, or other support were received during the preparation of this manuscript.

## 1 Introduction

Neurological disorders are recognized as a global health challenge, accounting for the leading cause of disability and second leading cause of death globally.^1^ The economic burden of neurological disease in the US alone is estimated to be approximately $765 billion.^2^ The application of wearable body sensors known as SmartWear in this patient population may play an integral role in improving outcomes and reducing healthcare costs.^3^^,^^4^ By continuously and remotely monitoring patients, SmartWear has the potential to decrease inpatient length of stay, a major contributor to cost in both stroke and neurosurgical patients.^5^^,^^6^ Additionally, these devices can improve medication adherence, optimize treatment plans, decrease the number of in-person physician visits, and increase patient engagement.^6^, ^7^, ^8^ As the use of SmartWear becomes more widespread, their adoption in the treatment of neurological and neurosurgical patients is critical to improving patient care and reducing healthcare costs associated with these conditions.

Despite the significant progress made in this field over the last decade, there is still a lack of literature regarding the use of SmartWear in neurological and neurosurgical diseases. In this paper, we provide a comprehensive overview of the types of clinical data measured by SmartWear and its use in patients with specific neurological conditions or requiring neurosurgical intervention. We also present up-to-date information on innovative technologies and ongoing clinical trials that demonstrate potential future applications of SmartWear. Finally, we address barriers to the implementation of SmartWear which must be overcome to further accelerate their widespread use in healthcare.

### 1.1 Application to neurological disease prevention and recovery

#### 1.1.1 Stroke

Globally, stroke is a leading cause of disability and mortality, and with an incidence of more than 795,000 per year in the United States alone, it imposes a significant burden on society.^9^, ^10^, ^11^ Timely intervention is crucial in the treatment of stroke and factors such as prehospital delay are a major barrier to receiving pharmacologic and surgical treatment.^10^, ^11^, ^12^ SmartWear has emerged as an easy-to-use and noninvasive tool to provide objective, quantifiable data for stroke surveillance and rehabilitation, with mounting evidence supporting its effectiveness.^13^^,^^14^ At the forefront are Zeit Medical's (Halo Alert System) wearable headband and the Neuralert wristband, which both use Electroencephalography (EEG) for stroke detection.^15^^,^^16^

These devices are being evaluated for use in high risk stroke patients, such as those with atrial fibrillation (AF).^17^^,^^18^ Kaisti et al conducted a study using a low-cost, wearable wristband designed for outpatient monitoring that showed favorable results in measuring heart rhythm and detecting irregularities using micro-electrical mechanical systems (MEMS).^19^ The device had almost identical pulse waveforms compared to the gold standard (invasive catheter recording) and effective classification accuracy between AF and sinus rhythm. More recently, the integration of machine learning algorithms and a wearable armband was able to continuously monitor for AF with high accuracy.^20^

While there is a paucity of evidence supporting screening asymptomatic carotid artery stenosis (10.13039/100005542CAS) for stroke prevention, targeted surveillance may be indicated in high-risk patients, such as those at risk for atheroma formation.^21^, ^22^, ^23^ A novel wearable carotid Doppler ultrasound neck patch (Foisonics Medical, Sudbury, Canada) showed favorable results in a study conducted with healthy volunteers.^24^ The patch was able to qualitatively and quantitatively follow blood flow metrics in the common carotid artery during both passive leg raise and quiet respiration. Its velocity measurement accuracy and ability to continuously record doppler spectrograms over many cardiac and respiratory cycles would prove useful in outpatient monitoring of high-risk CAS. The wearable Doppler patch can also minimize human measurement errors, monitor patients over extended periods, decreasing statistical limitations from inadequate beat sample size, all of which are limitations of handheld Doppler ultrasound.^25^^,^^26^ While the device is still in the proof-of-concept stage, it shows potential for use in outpatient stroke surveillance.

Evaluating motor impairments in stroke patients can be challenging due to variability among provider assessments and compensatory behavior from patients, however SmartWear is becoming increasingly popular in stroke rehabilitation to alleviate these issues.^27^, ^28^, ^29^, ^30^ Through, the use of Inertial measurement units (IMUs; measure kinetic variables) and Surface Electromyography (SEMG; measure muscle contraction), SmartWear has already been shown to identify and monitor hemi motor neglect, complex upper-limb movements, performance and posture, and whole-body tracking for patient evaluation and assessment of interventions like orthoses.^14^^,^^31^^,^^32^ Wei Chen et al reported 82 % and 90 % accuracy using a combined Apple watch (Apple, Cupertino, California, USA) IMU device to measure Activities of Daily Living (ADL) function in stroke patients based on ten and seven ADL tasks, respectively.^33^ Similarly, a randomized, parallel-controlled study of 120 motor impaired stroke patients investigated the use of remote rehabilitation with wearable IMUs attached to the affected arm, hand, thigh, and calf and demonstrated an increased motor ability with remote training with wearable IMU devices.^34^ Wearable SEMG devices can complement IMU data in motor assessment, and studies have shown that SEMG can enhance motor improvements, aid in diagnosis, and be used for outpatient stroke rehabilitation.^14^^,^^35^, ^36^, ^37^, ^38^, ^39^, ^40^, ^41^

The potential of SmartWear to make a significant impact on the management of stroke through surveillance, detection, and rehabilitation is clearly highlighted in the literature. Continued development of SmartWear, particularly for early detection, is critical to realizing the full potential of these devices in improving stroke outcomes.

#### 1.1.2 Epilepsy

Epilepsy is one of the most common neurological conditions, affecting approximately 50 million people globally.^42^ Timely recognition and response to seizures can play a pivotal role in improving outcomes and reducing long-term sequelae for patients.^42^, ^43^, ^44^ Currently, there are two devices that have been approved by the Food & Drug Administration (FDA) for use in epilepsy patients, the Empatica Embrace (EE) biosensor and the SPEAC surface electromyography (SEMG) system developed by Brain Sentinel.^45^^,^^46^ The EE is a band worn on the wrist or ankle that can detect body temperature, blood volume pulse, and motion via an accelerometer and electrodermal sensor.^45^ The EE can also detect preictal states and notify caregivers and/or patients via call or text. Clinical trials of the EE band demonstrated successful detection of 53 of 54 total generalized tonic-clonic seizures (GTCS) across 141 patients, including 80 children.^47^

The SPEAC system is a SEMG patch applied to the bicep that continuously monitors motor activity to detect convulsions.^48^ Like the EE band, this is also a cloud-based data platform that sends alerts to caregivers' phones, however in addition to detection it is also capable of characterization. Clinical validation of SEMG resulted in detection in 95 % of patients with high specificity.^49^ A characterization study then demonstrated the SPEAC system to have a 72 % accuracy rate when differentiating between tonic-clonic, tonic, clonic, and complex motor seizures as compared with an 81 % accuracy rate by epileptologists reviewing the same data.^50^ Although SPEAC is not as accurate, further development may improve accuracy of this system and can be used to aid in diagnosis.

Although there are few FDA approved devices, there are a multitude of other SmartWear devices that have been reported in the literature. In a 2017 multicenter prospective study, an armband assessing accelerometry and ECG signals was able to detect clinically urgent seizures with a sensitivity of 71–87 %.^51^ Although these results are positive, this study also reported a high number of false alarms. Similar to this, Halford et al demonstrated that a SEMG monitoring patch worn over the biceps muscle could detect 100 % of generalized tonic-clonic seizures, with a false alarm rate of 1.44 per 24 h and a positive predictive value of 6.2 %.^52^ More recently, Naganur et al used a wrist-worn MEMS accelerometer to detect all seizures occurring in a cohort of 11. Further, this system classified epileptic and psychogenic non-epileptic seizures with sensitivity and specificity of 72.7 % and 100 %, respectively. Similarly to the previous study, the authors reported a rate of 2.4 false alarms per 24 h, thus posing a potential drawback to these devices.^53^ As multiple studies have shown the potential for wearable devices in the detection of epilepsy, focus has now started to shift to improving the accuracy of these devices through the use of machine learning. Nasseri et al showed that machine learning models applied to data collected by the EE band, were able to forecast seizure alerts on average 33 min before the onset.^54^ Similar results have been reported, with decreases in false alarms, increased predictive accuracy and sensitivity becoming a common trend^55^, ^56^, ^57^

#### 1.1.3 Spinal cord injury

Annually, there are approximately 250,000 to 500,000 people who suffer from a spinal cord injury (SCI) worldwide.^58^ These patients have a high risk of medical complications due to motor deficits, disrupted regulation of bladder/bowel, cardiac, and/or respiratory functions, resulting in a decrease in functional improvement after discharge.^59^^,^^60^ Further, due to impaired activity and metabolic rate, patients with SCI are at increased risk for obesity which predisposes them to a multitude of health consequences.^61^ Therefore, the use of SmartWear to continuously monitor this population may improve outcomes and decrease preventable complications.

Loss of mobility makes regaining the ability to walk a key priority in the rehabilitation of patients with SCI; however, due to their injuries, these patients are also more likely to experience falls.^62^^,^^63^ This demonstrates the importance of remote monitoring using SmartWear technology. Lemay et al demonstrated through the use of a wearable IMU in SCI patients, that reliable and valid measurements of altered gait could be detected.^64^ Further, Noamani et al used accelerometers placed on the sacrum and tibia to identify changes in balance control after SCI.^65^ Although proof of concept, these studies are critical to the application of SmartWear for continuous monitoring of SCI patients to provide quantifiable data for optimizing rehabilitation and reducing falls.

Autonomic dysfunction is also common in SCI, with various manifestations and degrees of severity. Urinary dysfunction is one such symptom, causing both decreased quality of life and increased risk for severe infection.^66^^,^^67^ To address this issue, Fong et al proposed a wearable optical based sensor to continuously monitor bladder capacity and provide alerts to a mobile device.^68^ For direct modulation, Knight et al devised a novel wearable device, which when inserted into the anal canal, can use EMG to continuously monitor detrusor activity and stimulate nerves to modulate detrusor overactivity when detected.^69^ Although increased bladder capacity was achieved in a small cohort, side effects from stimulation (including autonomic dysreflexia) were observed. As bladder distension is a possible cause of autonomic dysreflexia, it is important to note that there is a lack of literature regarding the use of SmartWear in detecting this. Currently, Suresh et al.‘s use of a wrist-worn smart watch and machine learning model to accurately (94.10 %) detect early symptoms of autonomic dysreflexia remains one of the few studies demonstrating this application.^70^

Clinically, treatment of SCIs is complex and requires intensive monitoring to prevent further complications. Currently, the small body of literature regarding the use of SmartWear in SCI patients presents an important application of this technology, however there is a need for further studies.

#### 1.1.4 Neurodegenerative disease and essential tremor

As the elderly population continues to rise globally, the incidence of neurodegenerative diseases, such as Parkinson's Disease, are likely to follow this trend.^71^ Parkinson's Disease (PD) is the fastest growing neurodegenerative disease in the world, with an estimated 90,000 patients diagnosed per year in the US alone.^72^ Tremor is one of the earliest manifestations of PD, however, it is also a nonspecific symptom of idiopathic essential tremor (ET). SmartWear can be used to aid current methods of detection which are unable to detect subtle fluctuations and amplitude of tremors, leading to delayed diagnosis or misdiagnosis.^73^ Lopez-Blanco et al demonstrated that data collected from a smartwatch and smartphone could be used to accurately detect tremors that were slower than the human eye can confidently perceive.^74^ Further, when combined with machine learning, a wearable bracelet was able to detect tremors with an accuracy of 91.7 %.^75^ Technologies such as these can be used to quantitatively identify tremors remotely in high-risk patients.

Sleep disturbances are also a significant symptom in early PD and can greatly affect the quality of life as the disease progresses.^76^ McGregor et al, demonstrated the use of accelerometers placed on each wrist to measure sleep abnormalities can effectively recognize PD patients from non–PD controls.^77^ Further, use of smartphones paired with machine learning demonstrated accurate differentiation between patients with PD and idiopathic REM sleep behavior disorder.^78^

Symptom improvement in parameters such as tremors, falls, and the freezing of gait are often used to determine efficacy of PD treatment, however these are often difficult to monitor in an outpatient setting.^79^, ^80^, ^81^ In a 2016 study of PD patients, accelerometers placed on the back were used to measure various parameters of gait in a free-living setting, finding it to be more sensitive in determining symptoms of PD compared to a clinical setting.^82^ Following this study, Burq et al in 2022 demonstrated that the use of a smartwatch containing multiple sensors (gyroscope, IMU, etc) could remotely monitor PD patients with motor tasks and provide information regarding pharmacodynamic responses to dopaminergic medications.^83^ This and similar studies, suggest that SmartWear actively monitors response to therapies and provides data through continuous monitoring for the development of biomarkers for disease progression.^84^

SmartWear is also being used to detect and monitor other neurodegenerative diseases such as Alzheimer's and dementia.^85^^,^^86^ With established baseline efficacy of certain quantitative measurements (ex. Tremor amplitude pattern for PD) for diagnosis and/or management of these diseases, the use of SmartWear will serve as a valuable tool in providing earlier detection, accurate staging, and optimal management, especially when combined with machine learning techniques.^87^^,^^88^

#### 1.1.5 Recovery of neurosurgical patients post-operatively

Postoperative monitoring is a critical component of routine and high-risk neurosurgical care alike. While vital signs may provide important objective data in the immediate postoperative period, long-term outcomes of care are often described by subjective metrics such as Visual Analogue Score (VAS) and the Oswestry Disability Index (ODI). SmartWear enables providers to quantitatively understand parameters of patient recovery such as mobility, and posture, both of which are significant components to postoperative life.^89^, ^90^, ^91^

Reports of use in spinal stenosis, degenerative disc and spine disease, disc herniation, and spinal fusion procedures exist and these studies have found a correlation between the number of steps taken and improvement in patient outcomes. A 2019 study by Kim et al, was among the first to demonstrate the efficacy of using the popular Fitbit Charge (Google, San Francisco, California, USA) to measure the number of steps in postoperative laminectomy patients.^92^ The results of this study demonstrated a significant correlation between number of steps and improvement in both VAS and ODI scores. Similarly, the use of wearable accelerometers to measure step count, walking speed, step length and posture by Ghent et al, allowed for the establishment of a novel scoring tool which was positively correlated with change in ODI in lumbar surgery patients post-operatively.^93^ In regard to posture, Wang et al used a single tri-axis accelerometer amongst five patients with cervical spine injuries demonstrated 100 % accuracy in being able to classify different spinal postures.^94^ Moreover, smart garments and smart belts have shown promise in accurately determining improper posture and have even been shown to provide immediate feedback via vibratory pressure to users.^91^^,^^95^^,^^96^

Although much of the literature focuses on spinal surgery, these devices have been shown promise in other neurosurgical applications. In an endovascular application, a study analyzing postoperative carotid endarterectomy patients fitted with a belt containing an accelerometer demonstrated objective measurements in gait improvement post operatively.^97^ Additionally, evaluation of a smartwatch to monitor patients after transsphenoidal surgery found high adherence by patients and marked postoperative physiologic findings.^98^ Taken together, there is a clear role for SmartWear in the management of postoperative neurosurgical patients, however further evaluation of these devices in settings outside of spine surgery are warranted.

## 2 Discussion

**Fig. 1:**
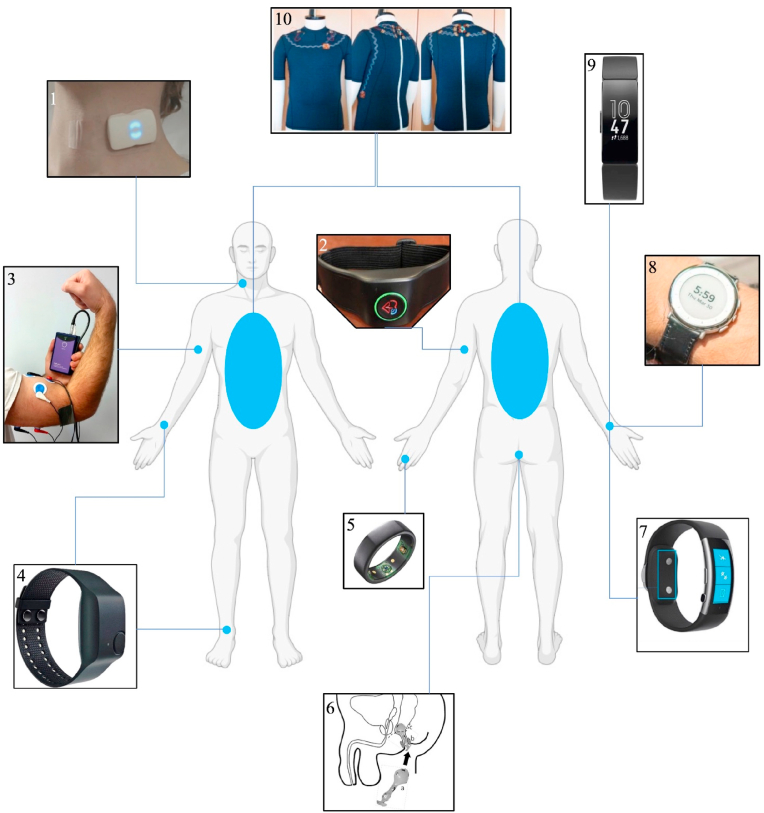
**Visual summary of SmartWear devices for neurological and neurosurgical applications.** Image 1 is a device used for continuous Doppler monitoring of the Carotid artery.^24^ Image 2 is a wearable armband for the detection of atrial fibrillation, a risk factor for stroke.^20^ Image 3 is Brain Sentinel's sEMG device worn on the biceps muscle for seizure monitoring/detection.^49^ Image 4 is the Empatica 4 which can be worn on the wrist or ankle for seizure monitoring/detection.^104^ Image 5 is the Oura ring which has been proposed for sleep monitoring in Alzheimer's disease.^85^^,^^105^ Image 6 is a neuromodulation device inserted into the anus for bladder control in patients with spinal cord injury.^69^ Image 7 is the Microsoft Band, which was used to detect autonomic dysreflexia in patients with SCI.^70^ Image 8 is smart wristwatch which can detect response to medication in PD patients.^84^ Image 9 is the FitBit Charge which was used to measure activity levels in patients post lumbar spinal fusion.^92^^,^^105^ Image 10 is a smart shirt which can detect postural changes.^91^ All image modifications have been cited and adaptations are protected by the Creative Commons license.

Recently, digital health and biosensor companies have spent billions of dollars, driving clinical trials and development of innovative SmartWear technologies.^99^ The increasing application of SmartWear technology in the clinical setting is also reflected in the literature as well, as summarized in Table 1 and Fig. 1, with large increases over the past two decades in both neurosurgery specific applications and medical applications overall.^100^^,^^101^

**Table 1:** Summary of SmartWear devices and applications.

Clinical Applications	Location	Type of Sensor	Marker
**Stroke**	Wrist	MEMS^19^	Arterial pressure waveform, arrhythmia detection, heart rate
	Neck	Doppler Ultrasound^24^	Carotid flow velocity
	Head	EEG^13^^,^^15^	Brain Activity
	Wrist	Accelerometer^16^	Asymmetric upper extremity weakness
	Wrist, Arm, Hip	Accelerometer, Gyroscope^33^	Motor activity
	Arm, Hand, Thigh, Calf	Accelerometer, Gyroscope, Magnetometer^34^	Motor activity
	Wrist or Ankle	Accelerometer, Electrodermal Sensor^47^	Body temperature, blood volume pulse, motion
	Arm	Electromyography^50^	Muscle activity
	Upper arm	Accelerometer and Electrocardiography^51^	Heart rate, acceleration
**Epilepsy**	Arm	Electromyography^52^	Muscle activity
	Wrist	Accelerometer^53^	Motor activity
	Wrist	Accelerometer, photoplethysmography, electrodermal activity, temperature^54^	Motor activity, blood volume pulse, electrodermal activity, heart rate, temperature
	Feet, Legs, and Sacrum	Accelerometer, Gyroscope^64^	Gait Characteristics
	Sacrum, Tibia	Accelerometer, Gyroscope^65^	Balance/Posture Control
**Spinal Cord Injury**	Anal Canal	Electromyography^69^	Muscle Activity
	Wrist	Electrodermal Sensor^70^	Galvanic skin response, heart rate, and skin temperature
	Wrist	Gyroscope^74^	Tremor (angular motion)
	Wrist	Accelerometer, gyroscope^75^	Tremor and bradykinesia detection (motor activity)
**Neurodegenerative Disease and Essential Tremor**	Wrist	Accelerometer^77^	Motor activity (used to calculate severity of bradykinesia)
	Back	Accelerometer^82^	Gait characteristics (motor activity)
	Wrist	IMU, gyroscope, photoplethysmography, skin conductance sensors^83^	Acceleration and angular momentum during specified tasks
	Wrist	Accelerometer, altimeter, vibration motor^92^	Ambulatory function, activity level
	Wrist	Accelerometers^93^	Step count, gait velocity, mean step length
	Head	Accelerometer^94^	Cervical curvature level
**Post-operative Recovery of Neurosurgical**	Back	Accelerometer^97^	Gait characteristics
	Wrist	Photoplethysmography, accelerometer, gyroscope^98^	Heart rate, heart rate variation, respiration, O2 saturation, calories, steps, distance
	Neck, clavicle^a^	Temperature^102^	Continuous monitoring of CSF flow
	Head^a^	Photoplethysmography^103^	Oxygenation, heart rate, cerebral pulse pressure, vascular tone

a Currently only tested for inpatient use.

A critical factor for the widespread adoption of these devices in healthcare is validation through clinical trials. Currently, there are multiple studies investigating the efficacy of SmartWear in patients with various neurological/neurosurgical diseases. Examples of these include a smartwatch application used to monitor medication adherence in patients requiring pharmaceutical intervention for secondary stroke prevention (NCT04180475) and a neuromodulator device worn on the foot/ankle to provide bladder modulation in those with overactive bladder syndrome (NCT05381116). The use of SmartWear in Parkinson's Disease specifically has seen an increase in trials for various applications, ranging from the use of a smartwatch to evaluate response to pharmacologic therapy (NCT05351580) to improving gait using a wearable cueing device (NCT04459559). These and similar technologies, represent the application of SmartWear for monitoring and treating certain neurological conditions. Finally, there are also clinical trials underway to investigate the use of SmartWear in broader applications (such as the Chronolife TM smart shirt to monitor cancer patients), which may be applied to neurological/neurosurgical patients.

From a neurosurgical perspective, the use of SmartWear has largely been focused on monitoring postoperative patients.^106^, ^107^, ^108^ Many of these studies have specifically investigated gait function in patients who have recently undergone spinal surgery.^106^^,^^102^, ^108^, ^109^ For example, Inoue et al, used a Micro-Motion logger system to determine the postoperative activity of sixty patients that recently underwent lumbar spinal surgery.^102^ The study found significantly lower activity one month postoperatively, but this improved at 3 months and 12 months postoperatively. While objective postoperative data on patient activity does provide some benefit, some studies in the literature do not show a significant relationship between physical activity and improvement in subjective clinical outcomes.^103^, ^110^, ^111^ It is important to note that many tests of clinical outcomes are subjective pain rating scores, and perhaps do not provide an accurate representation of a patient's recovery. Furthermore, Schulte et al, suggests that objective activity level is only one aspect of recovery and other factors such as motivation or postoperative symptoms may play a role in clinical improvement as well.^110^ The results of these studies suggest the need to control for many factors (type of surgery, age, motivation of patient, etc.) in order to determine the true relationship between objective activity level subjective clinical outcomes.

Although there are many studies of SmartWear use for spinal pathology, studies have shown feasibility for use in patients treated neurosurgically for essential tremors and deep brain stimulation.^112^^,^^113^ For example, Chockalingam et al, used a smartphone application known as Lift Pulse to measure essential tremors post ventralis intermedius thalamic deep brain stimulation.^113^ The study found a significant correlation between Lift Pulse improvement and Marin Tremor Rating Scale for arm tremor. Wearable devices used to monitor cerebrospinal fluid flow and pediatric cerebral hemodynamics are also being investigated in inpatient settings, representing a possible avenue for monitoring these vital parameters remotely.^114^^,^^115^ These pilot studies demonstrate the potential future applications of SmartWear to measure complex physiologic parameters allowing for reduced postoperative complications and comprehensive monitoring.

Alongside clinical validation, development of novel sensors for continuous monitoring of endogenous biomarkers has also accelerated.^116^ Examples of these novel devices include wrist-worn devices which can monitor/detect cortisol levels and self-powered optical devices (similar to contact lenses) to monitor hemodynamic vital signs.^117^, ^118^, ^119^ Although promising technology, there are many barriers which must be addressed as well, such as integrating the large volume of data generated by SmartWear. Concerns persist over data integration into current healthcare practices and electronic health record (EHR) systems, as well as data overload of physicians.^120^, ^121^, ^122^, ^123^ Large EHR companies such as Epic are working to provide solutions to these issues, linking their system directly with popular SmartWear devices, however there are still many devices which are excluded from this and data visualization for clinicians remains an issue.^122^^,^^124^

Furthermore, due to the wearable technology industry's rapid expansion, there is a lack of industry-wide standards for the transmission and encryption of health data.^121^^,^^122^^,^^124^, ^125^, ^126^ The inadequacies of security measures may result in the exposure of private health data, potentially damaging the trust essential to the doctor–patient relationship.^125^^,^^126^ Moreover, since the private sector is responsible for a majority of SmartWear innovation, providers have raised concern over inadequate regulatory oversight to ensure that health data is being used solely for the benefit of patients and not corporate profits.^122^^,^^125^^,^^127^ These concerns have already been realized with pharmaceutical companies paying physicians to target vulnerable populations for financial gain and the purchase of healthcare data by advertising giants such as Amazon, Google, and Microsoft.^128^

Beyond lack of regulatory oversight, this technology may also create further barriers for marginalized and underserved populations. Currently, the majority of health monitoring devices are used by insured patients, living in suburban/urban areas, who own smart wireless technology.^120^ Patients of lower socioeconomic classes and rural areas may have less access to Wi-Fi and other technologies and therefore also have limited access to SmartWear devices, despite being the individuals who may benefit most from them.^120^^,^^129^^,^^130^ Such inequalities can exacerbate existing health disparities (e.g. race and class-based disparities) in regards to both access and outcomes.^131^^,^^132^ Finally, although incorporating machine learning is critical to the development and integration of SmartWear, it may further aggravate health disparities due to the greater representation of patients from higher socioeconomic/educational backgrounds in the datasets used for training these models.^120^^,^^123^^,^^127^^,^^133^ This presents a significant sampling and selection bias as the health profiles of lower socioeconomic status and rural patients may not be weighed as heavily when using machine learning and SmartWear to guide clinical decisions. Thus, as SmartWear represents the future of personalized data driven healthcare, there are a multitude of ethical, legal, and practical considerations which must be addressed to become fully integrated into patient care (Table 2).

**Table 2:** Summary of merits and challenges/drawbacks of SmartWear devices and applications.

Benefits of SmartWear	Challenges and Drawbacks of SmartWear
Clinical	
- Non-invasive - Continuous monitoring of vital parameters - Improved quality of life - Increased patient empowerment/engagement - Remote medication adherence monitoring - Access to medical services (ex. rehabilitation) - Prevention and prediction (ex. seizures) - Post-operative assessment and decreased length of stay	-Accuracy concerns - Over-reliance by providers and patients - Compliance with device use
Technical	
- Ease of use - Many patients already own popular smart devices - Familiarity with many popular devices (ex. Smartwatches and smartphones) - Comfortable/discreet devices	- Some devices must be charged consistently - Software and hardware malfunctions - Need for data visualization software and integration into current electronic medical record systems - Regulatory oversight and standardized guidelines for secure transmission of private health data
Societal	
- Decreased number of in-person visits may improve healthcare reach for rural patients and/or low socioeconomic status	- Persons of low socioeconomic status may not be able to afford devices without insurance coverage - Data generated by devices may be prone to bias based on user population, thus skewing tools such as machine learning models - Protection of patient data from purchase for financial gain such as advertisements

## 3 Conclusion

The use of SmartWear is rapidly becoming adopted in the outpatient setting in order to monitor and treat neurological and neurosurgical patients. These devices can provide earlier detection, accurate diagnosis, precise treatment. The application of machine learning and artificial intelligence is critical to establishing predictive technology and improving sensitivity and specificity. Finally, although the future of SmartWear is promising, limitations such as integration and security must be addressed.

## CRediT authorship contribution statement

**Nithin Gupta:** Writing – review & editing, Writing – original draft, Visualization, Investigation, Data curation. **Varun Kasula:** Writing – review & editing, Writing – original draft, Investigation, Data curation. **Praveen Sanmugananthan:** Writing – review & editing, Writing – original draft, Investigation, Data curation. **Nicholas Panico:** Writing – review & editing, Writing – original draft, Investigation, Data curation. **Aimee H. Dubin:** Writing – review & editing, Writing – original draft. **David AW. Sykes:** Writing – review & editing, Writing – original draft. **Randy S. D'Amico:** Writing – review & editing, Supervision, Conceptualization.

## Declaration of competing interest

The authors have no relevant financial or non-financial interests to disclose.

## Abbreviations

ADL: Activities of Daily Living
AF: Atrial fibrillation
CAS: carotid artery stenosis
EEG: Electroencephalography
EHR: electronic health record
EE: Empatica Embrace
ET: essential tremor
FDA: Food & Drug Administration
GTCS: generalized tonic-clonic seizures
IMU: Inertial measurement units
MEMS: micro-electrical mechanical systems
ODI: Oswestry Disability Index
PD: Parkinson's Disease
SCI: spinal cord injury
SEMG: Surface Electromyography
VAS: Visual Analogue Score
